# Methyl donor supplementation reduces phospho‐Tau, Fyn and demethylated protein phosphatase 2A levels and mitigates learning and motor deficits in a mouse model of tauopathy

**DOI:** 10.1111/nan.12931

**Published:** 2023-08-28

**Authors:** Annika van Hummel, Goce Taleski, Jean‐Marie Sontag, Astrid Feentje Feiten, Yazi D. Ke, Lars M. Ittner, Estelle Sontag

**Affiliations:** ^1^ Dementia Research Centre, Macquarie Medical School, Faculty of Medicine, Health and Human Sciences Macquarie University Sydney New South Wales Australia; ^2^ School of Biomedical Sciences and Pharmacy University of Newcastle Callaghan New South Wales Australia; ^3^ Hunter Medical Research Institute New Lambton Heights New South Wales Australia

**Keywords:** Alzheimer's disease, folate, Fyn, methylation, one‐carbon metabolism, PP2A, Tau, tauopathy

## Abstract

**Background:**

Reduced folate status and elevated levels of circulating homocysteine are modifiable risk factors for cognitive decline and dementia. Disturbances in one‐carbon metabolism are associated with the pathological accumulation of phosphorylated tau, a hallmark feature of prevalent dementia, including Alzheimer's disease and subgroups of frontotemporal dementia.

**Methods:**

Here, using transgenic TAU58/2 mouse models of human tauopathy, we tested whether dietary supplementation with L‐methylfolate (the active folate form), choline and betaine can reduce tau phosphorylation and associated behavioural phenotypes.

**Results:**

TAU58/2 mice fed with the methyl donor‐enriched diet showed reduced phosphorylation of tau at the pathological S202 (CP13) and S396/S404 (PHF‐1) epitopes and alleviation of associated motor and learning deficits. Compared with mice on the control diet, the decrease in cortical phosphorylated tau levels in mice fed with the methyl donor‐enriched diet was associated with enhanced methylation of protein phosphatase 2A, the major brain tau Ser/Thr phosphatase. It also correlated with a reduction in protein levels of Fyn, a tau tyrosine kinase that plays a central role in mediating pathological tau‐induced neurodegeneration. Conversely, Fyn expression levels were increased in mice with deficiencies in folate metabolism.

**Conclusions:**

Our findings provide the first experimental evidence that boosting one‐carbon metabolism with L‐methylfolate, choline and betaine can mitigate key pathological, learning and motor deficits in a tauopathy mouse model. They give support to using a combination of methyl donors as a preventive or disease‐modifying strategy for tauopathies.

## Graphical abstract

Dietary supplementation with L‐methylfolate, choline and betaine boosted one‐carbon metabolism, resulting in enhanced PP2A methylation and decreased Fyn and phospo‐Tau levels, which slowed cognitive decline in a mouse model of tauopathy.

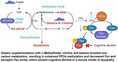

**Key Points**

- Dietary supplementation with L‐methylfolate (the active folate form), choline and betaine reduced cortical phosphorylated tau and alleviated cognitive and motor deficits in a TAU58/2 transgenic mouse model of human tauopathy.
- Methyl donor enrichment was associated with enhanced methylation of PP2A, the major brain tau Ser/Thr phosphatase.
- Methyl donor enrichment was associated with a reduction in protein levels of Fyn, a tau tyrosine kinase that plays a central role in mediating tau‐induced neurodegeneration.

## INTRODUCTION

While Alzheimer's disease (AD) is the most prevalent dementia, frontotemporal dementia (FTD) is the most common early‐onset dementia [1]. The clinical FTD syndrome is a heterogenous cluster of neurodegenerative disorders classified into broad subgroups according to pathological criteria; nearly half of FTD patients have a proteinopathy called frontotemporal lobe degeneration‐tau (FTLD‐Tau) [2]. The accumulation of hyperphosphorylated microtubule‐associated protein tau (p‐Tau) in neurofibrillary tangles and fibrillar aggregates is a pathological hallmark of AD and FTLD‐Tau [3]. Dysfunction of tau protein kinases [4], such as glycogen synthase kinase 3β (GSK3β), and phosphatases, including protein Ser/Thr phosphatase 2A (PP2A) [5], has been linked to abnormally increased phosphorylation of tau at pathological Ser/Thr epitopes. Aberrant epitope‐specific tau phosphorylation affects normal tau distribution and function, promoting tau dysregulation and aggregation [6]. Moreover, enhanced interaction of p‐Tau with Fyn, a major Src family tyrosine kinase (SFK), plays a critical role in mediating synaptic deficits and neurodegeneration in AD [7] and FTLD‐Tau [8]. Several mutations in *MAPT*, the tau‐encoding gene, are causal to familial FTD, further supporting the link between tau dysfunction and dementia [9].

Complex interactions between genetic and environmental factors likely trigger the onset of sporadic AD and FTD [10]. Of particular interest, reduced plasma folate and increased plasma homocysteine (Hcy) levels are independent modifiable risk factors for the development of cognitive decline, dementia [11], and AD [12, 13, 14]. Hyperhomocysteinaemia is also a risk factor for FTD [15]. Low serum folate levels correlate with cognitive deficits in FTD patients [16, 17]. Experimentally, folate deficiency and/or hyperhomocysteinaemia are associated with the development of pathological tau phosphorylation in cortical brain regions [18, 19, 20, 21, 22, 23, 24].

Folate and Hcy are key intermediates of one‐carbon metabolism, in which one‐carbon (methyl) units are derived from nutrients (via food intake or supplements) to be utilised for fundamental cellular processes, such as methylation, biosynthesis and redox reactions [25]. One‐carbon metabolism comprises interlinked folate and methionine cycles (see graphical abstract). The biologically active folate species, 5‐methyltetrahydrofolate (5‐MTHF or methylfolate), plays an essential role in one‐carbon metabolism. It is ultimately generated in the folate cycle from dietary folate via the action of the rate‐limiting enzyme, 5,10‐methylenetetrahydrofolate reductase (MTHFR). 5‐MTHF is the primary provider of one‐carbon units for the remethylation of Hcy to methionine in the methionine cycle. Other important one‐carbon nutrients, including betaine and its precursor, choline, can be used in a parallel pathway to remethylate Hcy to methionine [26]. Methionine is essential for the generation of the universal methyl group donor, S‐adenosylmethionine (SAM). During methyl transfer, SAM is converted to S‐adenosylhomocysteine (SAH), which is hydrolysed to Hcy. Hcy can be metabolised via a transsulphuration pathway that is critical for the production of cysteine‐containing amino acids and the synthesis of glutathione (GSH), a crucial cellular antioxidant [25].

Disturbances in one‐carbon metabolism, including dietary and genetic deficiencies in folate metabolism, can lead to abnormal and cytotoxic elevation of Hcy [11]. Elevated Hcy levels promote oxidative stress and excitotoxicity [11] and irreversibly modify proteins, including tau [27]. They lead to the accumulation of SAH, an inhibitor of SAM‐dependent methyltransferases [11]. Reduced blood GSH and SAM levels, indicating altered redox state and methylation capacity, are associated with cognitive decline, dementia and AD [28, 29]. Methyl‐donor nutrients are crucial to neurodevelopment and brain health throughout life and have been used in clinical trials of cognition in healthy adults with mixed results (reviewed in McKee and Reyes [30]). SAM levels are decreased in AD cerebrospinal fluid (CSF) [31] and brain tissue [32]. Increased SAH and decreased 5‐MTHF in the CSF are strongly associated with cognitive impairment and CSF p‐Tau levels in AD [33].

Notably, alterations in folate and Hcy metabolism impair PP2A catalytic subunit (PP2Ac) methylation [18, 19, 20, 21, 22]. PP2Ac methylation promotes the formation and stabilisation of Bα subunit containing PP2A holoenzymes, the predominant brain tau Ser/Thr phosphatases [34]. The accumulation of demethylated PP2A species occurs in AD [35] and FTLD‐Tau [36] affected brain regions and correlates with enhanced phosphorylation of tau at AD‐like epitopes in cells and in vivo [5, 34].

We have previously demonstrated that the P301S mutant tau transgenic TAU58/2 mouse model recapitulates many features of AD and FTLD‐Tau. TAU58/2 mice develop progressive early‐onset motor deficits, behavioural abnormalities and axonal pathology from 2 months of age and p‐Tau pathology from 3 months of age, which becomes more abundant over time [37, 38, 39, 40]. Based on the strong link between deficiencies in folate and Hcy metabolism and AD/FTD, we tested here the hypothesis that dietary supplementation with L‐methylfolate, choline and betaine could mitigate p‐Tau pathology and behavioural deficits in TAU58/2 mice.

## MATERIALS AND METHODS

### Reagents and antibodies

Unless indicated, all chemicals were purchased from Sigma/Merck Millipore. Antibodies used for immunoblotting included: mouse anti‐demethylated PP2Ac (clone 1D6, Merck Millipore); mouse anti‐PP2Ac_α/β_ (clone 46, BD Transduction); rabbit anti‐phospho‐p44/42 mitogen activated protein kinase (MAPK) (#9101, Cell Signaling Technology); mouse anti‐p44/42 MAPK (ERK1/2) (L34F12, Cell Signaling Technology); rabbit anti‐phospho‐GSK3β (Ser9) (D85E12, Cell Signaling Technology); mouse anti‐GSK3β (clone 3D10, Cell Signaling Technology); rabbit anti‐Tau (T‐1308‐1, rPeptide); mouse anti‐pS202‐Tau (CP13) [41] and anti‐pSer396/Ser404 (PHF‐1) tau (kindly donated by Peter Davies); mouse anti‐pS422‐tau (44‐764G, ThermoFisher); mouse anti‐human Tau‐13 (ab19030, Abcam); rabbit anti‐Fyn (clone EPR5500, Merck Millipore); mouse anti‐Fyn clone 25 (BD Transduction Laboratories); rabbit anti‐phospho‐SFK (Y416) (clone 100F9, Cell Signaling Technology); mouse anti‐actin (clone C4, Merck Millipore); and mouse anti‐glyceraldehyde 3‐phosphate dehydrogenase (clone 6C5, Merck Millipore). Mouse anti‐Fyn (clone 15, Santa Cruz), rabbit anti‐pS202‐Tau (ab108387, Abcam) and PHF‐1 antibodies were used for immunostaining.

### TAU58/2 mice and diet treatment

TAU58/2 mice expressing the human 0N4R tau isoform with the P301S mutation under the control of the mouse Thy1.2 promoter have been thoroughly characterised [37, 38]. Mice were maintained heterozygous on a C57BL/6 background and housed in filter top cages (maximum of five mice per cage) containing nesting material, a wooden stick and a transparent red dome and maintained on a 12 h light/dark cycle with food and water ad libitum. All experiments were conducted with female mice due to the later onset and slower progression of phenotypes in female compared with male TAU58/2 mice [37]; this allowed us to test the potential beneficial effects of a short‐term dietary intervention prior to the onset of irreversible neurodegeneration. Because our study aimed to specifically test whether supplementation with methyl donors can ameliorate p‐Tau pathology and associated symptoms and given that wild‐type (WT) C57BL/6 mice do not develop p‐Tau pathology, those were not included in this study.

TAU58/2 mice were weaned at 3 weeks of age onto either a custom L‐amino acid‐defined rodent diet (A16012901, Research Diets Inc, NJ, USA) supplemented with 30 mg/kg L‐methylfolate™ (Merck), 9.09 g/kg choline bitartrate, 5 g/kg betaine and 0.5% ascorbic acid (to prevent L‐methylfolate oxidation) or the corresponding nutrient/calorie‐matched control diet lacking these supplements (A10021, Research Diets Inc), 12 mice per diet group. Mice were maintained on this diet for the duration of the experiment. All animal experiments were approved by the Animal Ethics Committees of Macquarie University. All procedures complied with the statement on animal experimentation issued by the National Health and Medical Research Council of Australia.

### Behavioural, memory and motor testing

All tests were performed in TAU58/2 mice (12 mice per diet group) 3 months after the start of the dietary intervention. Testing and analysis of videos were performed blinded to diet.

#### Pole test

To test strength and coordination, mice were placed at the top of a vertical pole (47.5 cm length of dowel, 0.8 cm diameter) facing upwards and allowed to turn and descend [42]. The best time out of two turns taken to turn and descend was chosen. Mice unable to complete the test (slipped or fell) were given the maximum score of 120 s.

#### Beam challenge

To assess sensorimotor deficits, mice were placed at one end of a horizontal pole (47.5 cm length of dowel, 0.8 cm diameter) suspended over a Perspex box, and a nesting house was placed at the far end to motivate mice to cross [37]. The best time taken to cross was recorded over two sessions, and mice who failed to cross within the maximum time (2 min) or fell off were excluded.

#### Morris water maze

To assess spatial learning and memory, the Morris water maze was conducted as previously described [39]. The apparatus consisted of a 1.5 m diameter tank with a 40 cm high Perspex platform (10 cm diameter) which was placed ~20 cm from the wall edge, with four visual cues of different shapes placed around the tank. The tank was filled with water and non‐toxic acrylic white paint to a height of 0.5–1 cm above the platform. Days 1–5 consisted of an acquisition phase, in which mice were placed at one of four starting positions in the opposite quadrant to the platform and given 60 s to find the platform. Mice that failed were guided to and remained on the platform for an additional 60 s before being removed from the maze. Mice had four consecutive trials with alternating starting positions each day. On the sixth day, the platform was removed, and mice were given 30 s to explore (probe trial). On the seventh day, the platform was placed back into the pool, and visual cues were removed to ensure all mice had normal vision. Videos were analysed using AnyMaze software (Stoelting Co, IL, USA), and trace plots were obtained for each trial during the learning phase. Individual trace plots were visually classified according to published criteria [43] and scored as follows: 1—thigmotaxis; 2—random swim; 3—scanning; 4—chaining; 5—directed search; 6—focal search; or 7—direct swim. These were then categorised as hippocampal (strategies 4–7) or nonhippocampal (strategies 1–3) dependent.

#### Elevated plus maze

Anxiety and disinhibition‐like behaviour were assessed on the elevated plus maze as previously described [42]. Briefly, mice were placed on the centre platform (5.5 cm × 5.5 cm) of a maze consisting of two closed and two open arms (each 35 cm × 5.5 cm) elevated 60 cm off the ground (Ugo Basile) and recorded for 5 min. Videos were analysed using AnyMaze software.

#### Open field

Activity, anxiety and exploration patterns were assessed as previously described [42]. Briefly, mice were placed at the periphery of a 40 cm × 40 cm Perspex box in an enclosed cupboard and recorded for 10 min. Videos were analysed using AnyMaze software, and the box was divided into an outer and inner zone (17.5 cm × 17.5 cm square in the centre of the box).

### Tissue collection

At 5 months of age, following completion of all behavioural tests, mice were anaesthetised (125 mg/kg ketamine + 25 mg/kg xylazine intraperitoneal [IP]), terminal blood was collected via cardiac puncture and then mice were transcardially perfused with phosphate‐buffered saline (PBS). The brain was removed, and the hemispheres were separated. One hemisphere was immersion‐fixed overnight in 4% paraformaldehyde for immunohistochemical analysis; the other was subdissected and snap‐frozen in liquid nitrogen for biochemical analysis. Total blood was collected in ethylenediaminetetraacetic acid (EDTA) tubes (Sarstedt, Germany) and centrifuged at 4°C for 10 min at 2000 ×*g* to obtain plasma.

### Plasma metabolite analyses

Plasma samples (*n* = 6 per diet group) were sent to Creative Proteomics (Shirley, NY, USA) for triplicate analyses by high‐performance liquid chromatography‐mass spectrometry (HPLC‐MS)/MS (Agilent 1290 UHPLC coupled to a Sciex 4000 QTRAP mass spectrophotometer operated in multiple‐reaction monitoring mode with negative ion (−) or (+) ion detection) of key metabolites of one‐carbon metabolism according to their protocols (see Supporting Information S1 for detailed protocols).

### Histological analyses

Brain tissue sectioning and staining were performed as described previously [44]. After overnight fixation in 4% paraformaldehyde, brain tissue was changed into 70% ethanol before being placed into histology cassettes and processed overnight in the Excelsior tissue processor (ThermoFisher, MA, USA). Processed tissue was embedded in paraffin, and 3 μm sections were cut on an HM 355S Automatic Microtome (ThermoFisher, MA, USA). Sections were air‐dried overnight. Before the staining procedure started, slides were baked for 2 h at 65°C (*n* = 6 per diet group). Residual paraffin was removed by placing slides in Xylene for 20 min after which they were rehydrated in decreasing concentrations of ethanol, ending in water. Heat‐mediated antigen retrieval was performed in a Milestone Histology microwave (Milestone, Sorisole, Italy) using citric buffer. Sections were stained using sequenza racks (ThermoFisher, MA, USA). Slides were blocked in 100 μL blocking buffer (BB; PBS containing 3% heat‐inactivated goat serum and 2% bovine serum albumin) for 1 h at room temperature. Slides were incubated overnight at 4°C with 100 μL of primary antibody diluted in BB (PHF‐1 1:250; anti‐Fyn 1:250; and anti‐pS202‐Tau 1:100), washed with PBS and then incubated for 1 h at room temperature with 100 μL of AlexaFluor‐conjugated 488 or 555 secondary antibodies (1:250, Invitrogen, Carlsbad, California, United States) and DAPI (1:1000, Molecular Probes, Carlsbad, Ca, USA) diluted in BB. No‐antibody controls and nontransgenic control tissue were included in each round of staining to ensure antibody specificity. Slides were washed and mounted using Fluoromount‐G (SouthernBiotech, Birmingham, AL, USA). Once dried, slides were scanned using an Axioscan Z.1 slide scanner (Zeiss, Oberkochen, Germany) with a 20× objective and narrow‐band filters (412–438, 501–527 and 585–675). Quantification was done by an investigator blinded to the experimental groups. Fluorescence intensity was quantified across the entire cortical region using in‐built Zeiss Zen 2.6 Blue software tools and normalised to area, and cell counts were performed manually using Zeiss Zen 2.6 Blue software or semiautomated using ImageJ with macros, with set parameters for each antibody.

### Western blot analyses

Cortical tissue homogenates from TAU58/2 mice (*n* = 6 per diet group) were prepared exactly as described previously [45] and analysed for p‐Tau as published earlier [18, 19]. As indicated, Western blot analyses were also performed using cortical tissue homogenates from 5 week old WT *Mthfr* ^+/+^, heterozygous (HET) *Mthfr* ^+/−^ and homozygous (NULL) *Mthfr* ^−/−^ female mice fed with a normal chow diet (*n* = 6 per genotype) that were obtained from our earlier studies [22, 45].

Aliquots (∼60 μg proteins per lane) were separated on NuPAGE™ Novex™ Bis‐Tris 4–12% gradient gels (ThermoFisher Scientific) and transferred overnight onto nitrocellulose membranes. Precision Plus Protein™ Standards (BIO‐RAD) were used as molecular weight standards. Western blotting was performed using the indicated primary antibodies, followed by *Infrared* IRDye®‐labelled secondary *antibodies* and visualisation using the Odyssey™ Infrared imaging system (LI‐COR Biosciences), as described previously [45, 46]. Band intensity was determined by two separate investigators using Image Studio Lite version 5.2.5 Software (LI‐COR Biosciences) to ensure accurate quantification of protein expression levels. Anti‐actin and anti‐glyceraldehyde 3‐phosphate dehydrogenase antibodies were used to normalise for protein loading. Protein demethylation or phosphorylation levels were determined after normalisation for total protein expression levels [46].

### Statistical analysis

All statistical analyses were done using the GraphPad Prism 9 software. All analyses were performed using a Student's *t*‐test, except for the pole test time (Mann–Whitney), Morris water maze swim path and pole test completions (binomial distribution) and the Morris water maze learning, elevated plus maze time in area, open field time in area and distance travelled (two‐way ANOVA). *P* values < 0.05 were considered significant. Sample sizes were informed by previous studies in TAU58/2 mice [37, 38, 39, 40].

## RESULTS

### Dietary supplementation with methyl donors ameliorates motor and learning deficits in TAU58/2 mice

**FIGURE 1:**
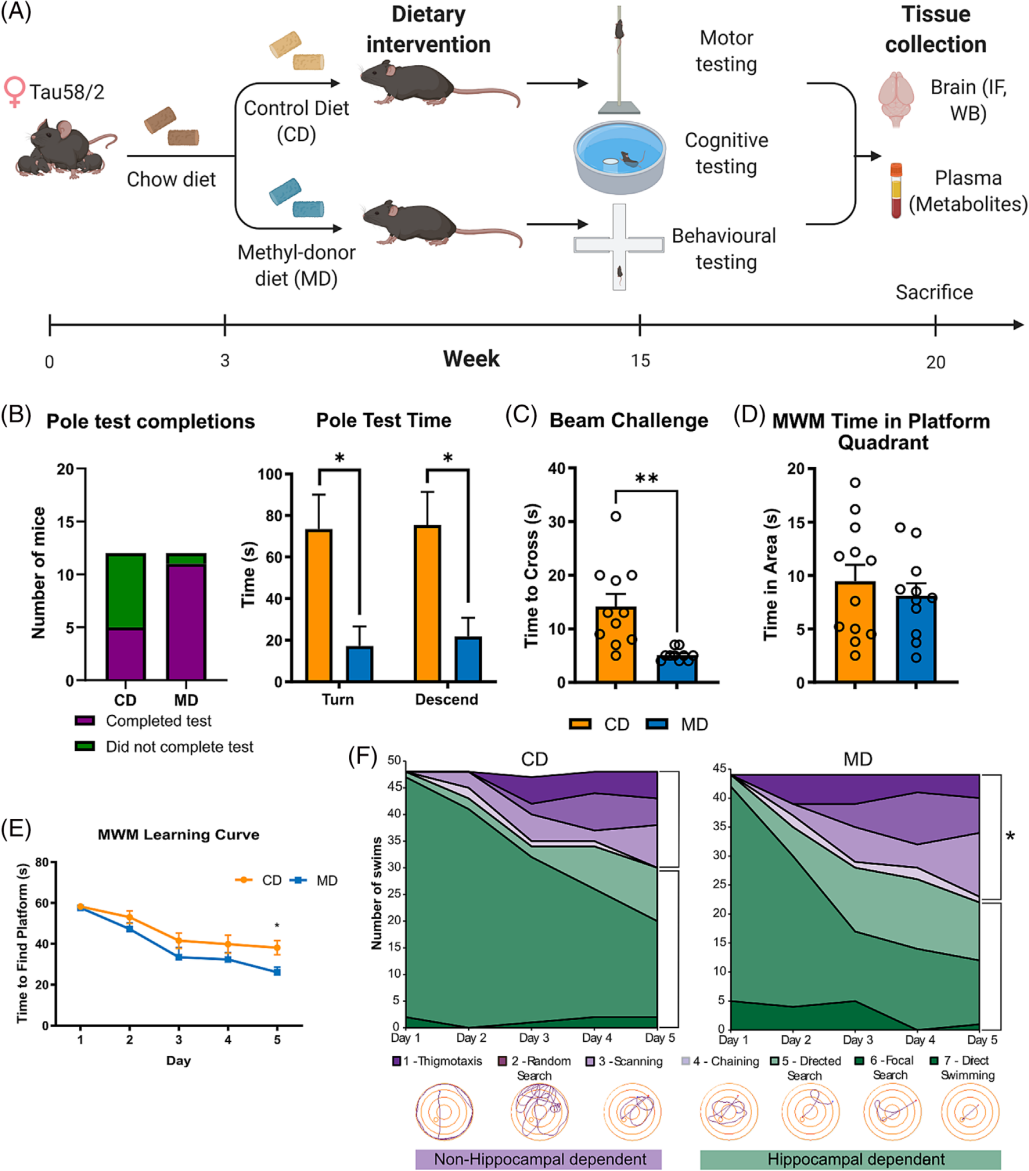
Dietary supplementation with methyl donors rescues Tau‐induced motor and cognitive deficits in TAU58/2 mice. (A) Three week old female TAU58/2 transgenic mice were placed on a methyl donor diet (MD) or the control diet (CD). Mice underwent behavioural testing at 3 months and were sacrificed at 5 months of age, with plasma and brain tissue collected for biochemical analyses. Diagram adapted from a template by BioRender.com (https://app.biorender.com/biorender‐templates/t‐5fd2691636507f00a448c50f‐mouse‐high‐fat‐diet‐experimental‐timeline). (B) (left) Number of mice who successfully completed the pole test and (right) time to turn and descend the pole test; Mann–Whitney test, *p < 0.05. (C) Time to cross the beam challenge; *t*‐test, **p < 0.01. (D) Time spent in the platform quadrant during the memory consolidation (probe trial) phase of Morris water maze (MWM) testing; *t*‐test, *p* > 0.05, MD vs CD. (E) Time to find the platform in the learning acquisition phase; two‐way ANOVA (*F* = 4.89, *p* = 0.0382); *p < 0.05, MD vs CD. (F) Swim path strategies used by mice in the learning acquisition phase in CD‐ (left) and MD‐fed (right) mice. The relative use of nonhippocampal dependent (thigmotaxis, random search and scanning) vs hippocampal‐dependent (visually driven chaining response, directed search, focal search and direct swimming) swimming strategies is shown. Data were appraised using the binomial distribution, *p < 0.05, MD‐ vs CD‐fed mice. Data in (B)–(E) are mean ± standard error of the mean (SEM), *n* = 12 mice per diet group.

Female TAU58/2 transgenic mice first underwent cognitive, motor and behavioural testing 3 months after being fed with either a methyl donor diet supplemented with L‐methylfolate, betaine and choline (MD) or the corresponding control diet (CD) (Figure 1A). Motor performance was significantly improved in MD‐fed mice on the pole test, with nearly all mice (~92%) successfully completing the test compared with only ~42% of CD‐fed mice (Figure 1B, left; *p* = 0.0005), and shorter time to turn (*p =* 0.0168) and descend (*p =* 0.0190) (Figure 1B, right). MD‐fed mice also performed better on the beam challenge (Figure 1C), with a shorter time to cross (*p* = 0.0015). During Morris water maze testing, memory consolidation was not affected by either diet, as shown by time in the platform quadrant during the probe trial (Figure 1D). However, MD‐fed mice showed faster learning acquisition, which was significantly improved at Day 5 (*p* = 0.0110; Figure 1E), with no difference in swim speed to account for the differences (Figure S1D). Assessment of the swim strategies showed a significantly larger proportion of swims on Days 2 (*p* = 0.0218), 4 (*p* = 0.0307) and 5 (*p* = 0.0289) being hippocampal vs nonhippocampal dependent in MD, compared with CD‐fed mice (Figure 1F). In contrast, mice in the MD and CD groups performed similarly in the elevated plus maze and open field tests that assess anxiety and disinhibition‐like behaviour (Figure S1).

### Dietary supplementation with methyl donors reduces cortical phosphorylated tau levels in TAU58/2 mice

**FIGURE 2:**
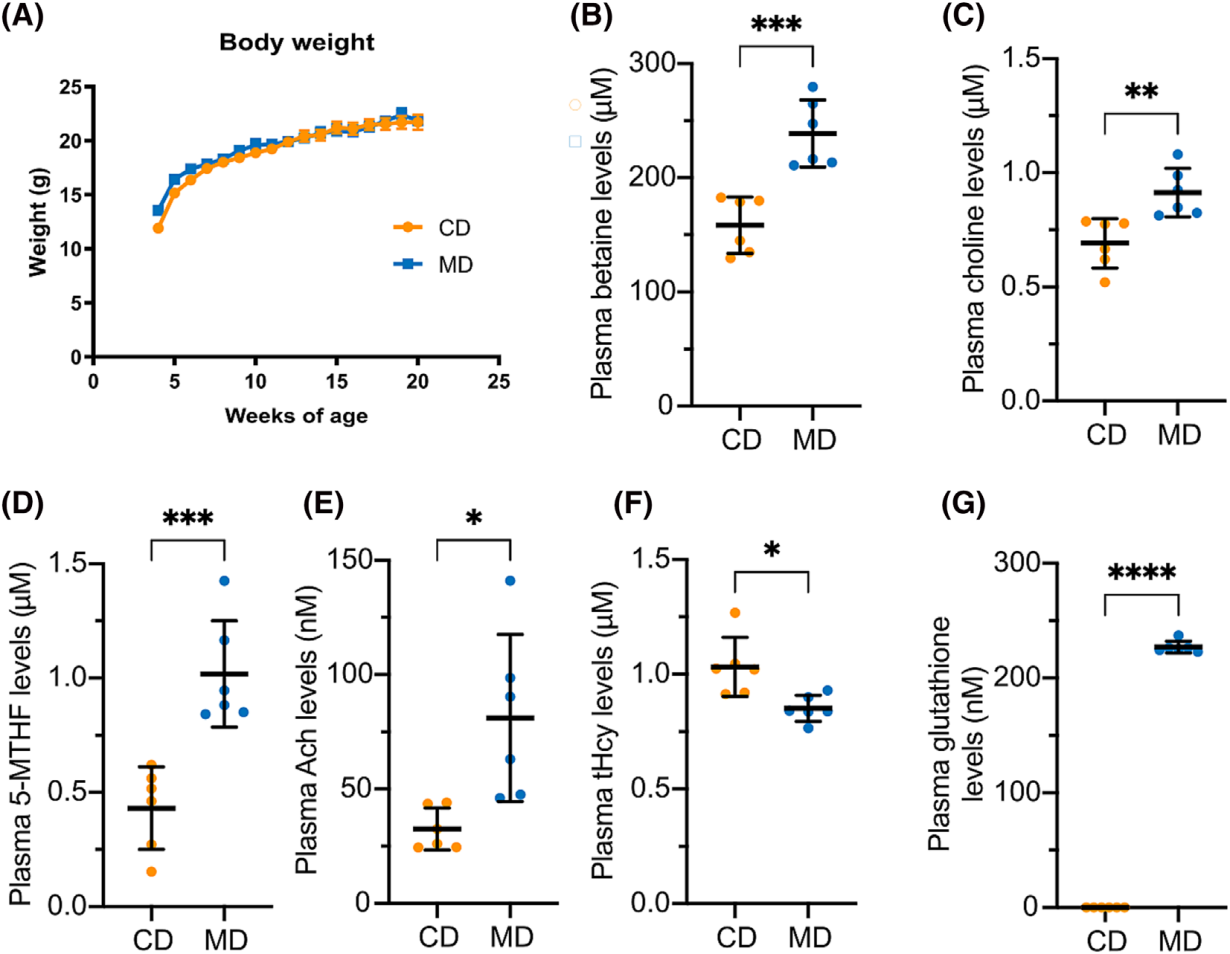
Impact of dietary supplementation with methyl donors on one‐carbon metabolism in TAU58/2 mice. (A) TAU58/2 mice were weighed weekly after the commencement of the control diet (CD) and methyl donor diet (MD). Data are mean ± SEM, *n* = 6 mice per diet group. There were no differences (*t*‐test, *p* > 0.05) in the weight of MD‐ vs CD‐fed mice. (B–G) Mouse plasma samples were analysed for betaine (B), choline (C), 5‐methyltetrahydrofolate (5‐MTHF) (D), acetylcholine (Ach) (E), total plasma homocysteine (tHcy) (F), and glutathione (G). Data in (B)–(G) are mean ± standard deviation (SD), *n* = 6 mice per group; *t*‐test, *p < 0.05, **p < 0.01, ***p < 0.001, ****p < 0.0001.

We first verified that our dietary intervention did not induce any adverse metabolic effects, as evidenced by equivalent body weights of mice in MD and CD groups over the course of the experiment (Figure 2A). Next, key compounds of one‐carbon metabolism were comparatively analysed in plasma samples from MD‐ and CD‐fed mice to confirm that our custom diet exerted its intended effects. Expectedly, there was a significant increase in basal plasma levels of betaine (~1.5‐fold; *p* = 0.0004; Figure 2B), choline (~1.3‐fold; *p* = 0.0049; Figure 2C) and 5‐MTHF (~2.4‐fold, *p* = 0.0006; Figure 2D) in MD‐fed mice, demonstrating proper absorption of these nutrients. In agreement with choline being a precursor to the major neurotransmitter, acetylcholine (Ach), circulating levels of Ach were increased by ~2.5‐fold (*p* = 0.0101) in MD‐fed mice (Figure 2E).

Total plasma Hcy (tHcy) levels in the TAU58/2 mice on the CD diet were below the threshold for mild hyperhomocysteinaemia [47], as previously reported in AD mice fed with a normal diet [48]. Notably, basal tHcy levels were decreased by ~20% (*p* = 0.0106) in MD‐ vs CD‐fed mice (Figure 2F). Plasma GSH levels were below detectable levels in the CD group but significantly elevated (*p* < 0.0001) in the MD cohort (Figure 2G). Increased levels of this antioxidant and decreased circulating tHcy levels are consistent with enhanced metabolism of Hcy via the transsulfuration pathway.

**FIGURE 3:**
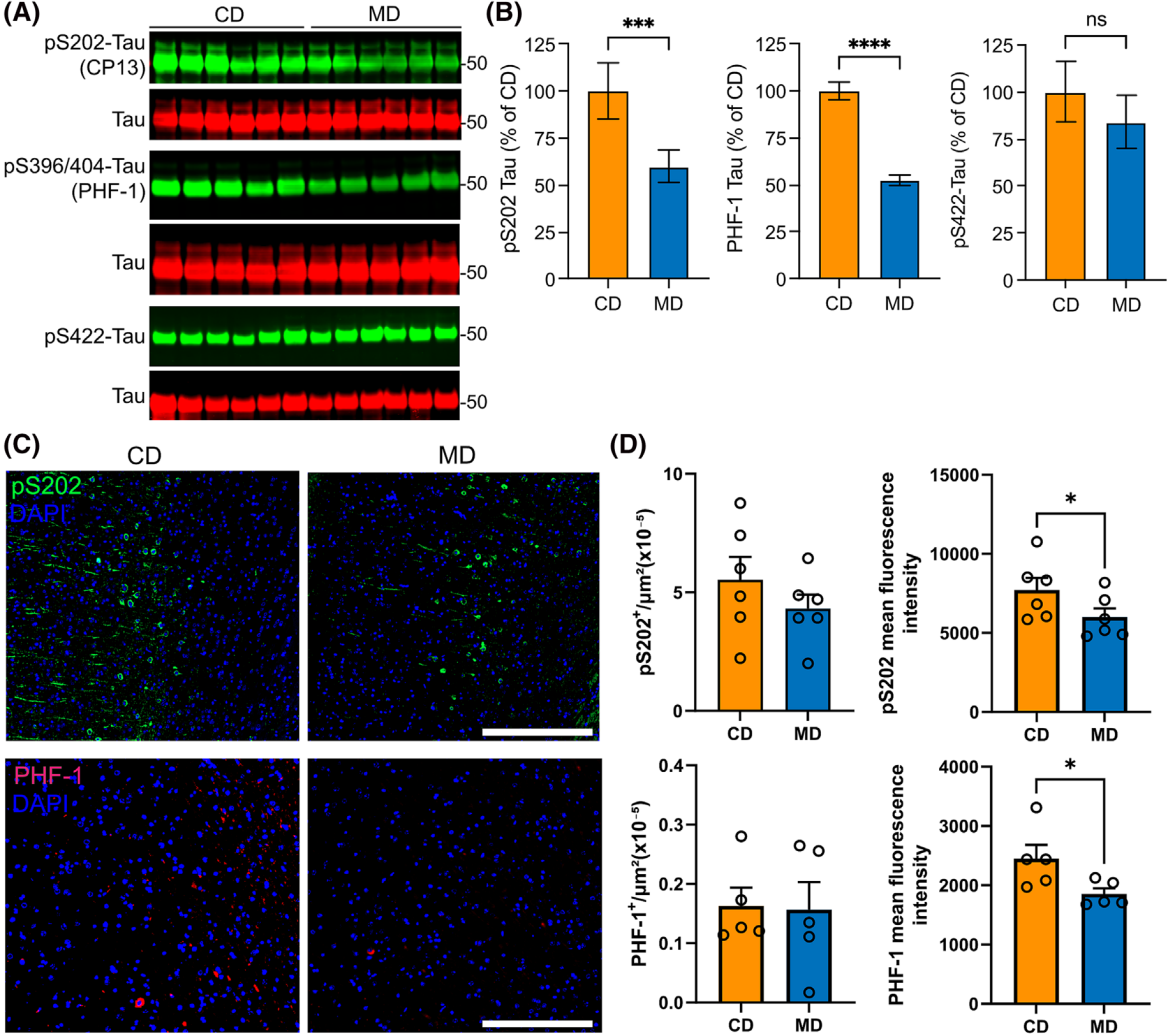
TAU58/2 mice fed with the methyl donor diet (MD) have reduced cortical Tau phosphorylation at pathological CP13 and PHF‐1 epitopes. (A) Western blot analyses of cortical homogenates from control diet‐ (CD) and MD‐fed TAU58/2 mice for pS202‐Tau (CP13 antibody), pS396/404‐Tau (PHF‐1 antibody), pS422‐Tau and total tau. (B) Quantification of pS202‐Tau (left), PHF‐1‐Tau (middle) and pS422‐Tau (right) levels. Data are mean ± SD, *n* = 6 mice per group; *t*‐test, ***p < 0.001, ****p < 0.0001; ns, not significant. (C) Representative cortical sections from CD‐ and MD‐fed TAU58/2 mice labelled with anti‐pS202‐Tau and PHF‐1 antibodies and counterstained with DAPI for nuclei. Scale bars, 50 μM. (D) Quantification of total cell number normalised to the area (left) and mean fluorescence intensity (right) for pS202 (top) and PHF‐1 (bottom). Data are mean ± SEM, *n* = 6 mice per group; *t*‐test, *p < 0.05, MD vs CD.

We next assessed the effects of the diet on tau phosphorylation at pathological epitopes in the cortex of TAU58/2 mice. Western blot analyses of cortical tissue homogenates showed a ~40–50% reduction in the levels of tau phosphorylated at the CP13 (pS202; *p* = 0.0002) and PHF‐1 (pS396/S404; *p* < 0.0001) epitopes in MD relative to CD‐fed mice (Figure 3A,B). Likewise, immunofluorescence analyses of cortical p‐Tau revealed that, although there was no difference in cell number, there was a significant decrease in the mean fluorescence intensity for pS202 (~0.2‐fold; *p* = 0.0489) and PHF‐1 (~0.2‐fold; *p* = 0.0238) in the MD, compared with the CD‐fed cohorts (Figure 3C,D). In contrast, there were no detectable changes in tau phosphorylation at the pS422 or pS214 sites (Figures 3B and S2); the minimal phosphorylation at these sites in 3 month old female TAU58/2 mice [37] may explain these results.

### Dietary supplementation with methyl donors reduces levels of demethylated PP2A

**FIGURE 4:**
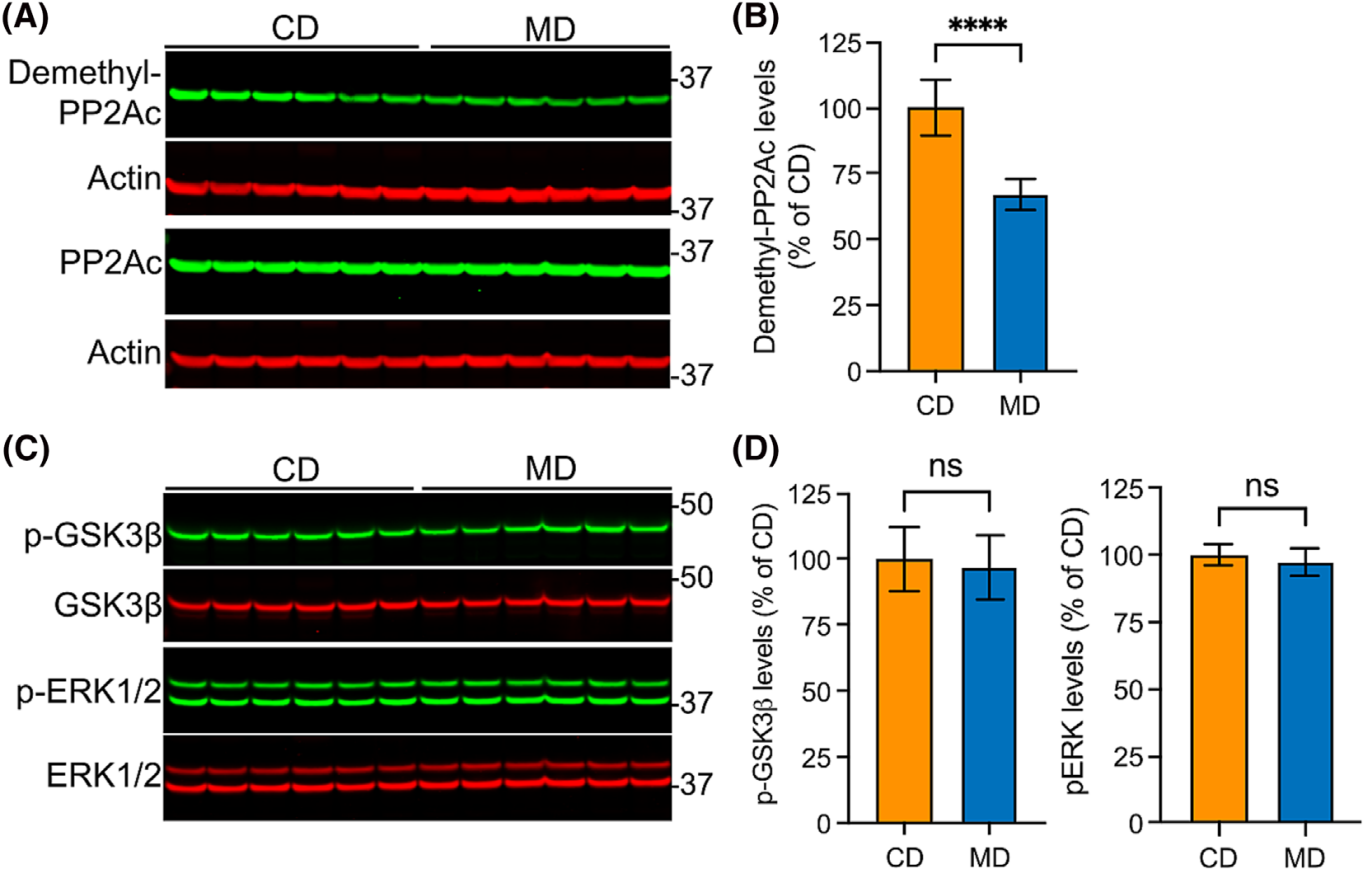
Dietary supplementation of TAU58/2 mice with methyl donors decreases cortical levels of demethylated PP2A without affecting ERK and GSK3β. (A) Cortical homogenates from control diet‐fed (CD) and methyl donor diet‐fed (MD) TAU58/2 mice were analysed by Western blot using an anti‐demethyl‐PP2Ac antibody specifically recognising demethylated PP2Ac species (Demethyl‐C, upper panel) and a methylation‐insensitive anti‐PP2Ac antibody recognising total PP2Ac (PP2Ac, lower panel). Actin antibodies were used to control for protein loading. (B) Quantification of demethylated PP2Ac levels. (C) Aliquots of the same homogenates from (A) were analysed by immunoblotting for active, phosphorylated ERK (p‐ERK) using validated antibodies recognising the phosphorylated forms of ERK2 (or p42‐MAPK, lower band) and ERK1 (or p44‐MAPK, upper band), inactive GSK3β phosphorylated at the inhibitory S9 site (p‐GSK3β) and total ERK1/2 and GSK3β. (D) Quantification of p‐GSK3β (left) and p‐ERK (right) levels. Data in (B) and (D) are mean ± *SD*, *n* = 6 mice per group; *t*‐test, ****p < 0.0001; ns, not significant.

Methylated PP2A holoenzymes play a major role in dephosphorylating tau at many Ser/Thr phosphoepitopes, and increased PP2A demethylation is associated with the accumulation of p‐Tau [5, 34]. Because the PP2A methylation state is dependent on the methylation cycle [18], we explored whether the decrease in p‐Tau levels in MD‐fed mice was mediated by a reduction in endogenous demethylated PP2A levels. Western blot analyses of cortical tissue homogenates showed that demethylated PP2Ac levels were decreased by ~33% (*p* < 0.0001) in MD compared with CD‐fed mice (Figure 4A,B). In contrast, the diets did not affect the phosphorylation (a readout of kinase activity) of the two major tau Ser/Thr protein kinases [4], GSK3β and ERK (Figure 4C–E). These findings suggest that reduced PP2A demethylation—and therefore, enhanced PP2A methylation—promotes the dephosphorylation of p‐Tau in MD‐fed TAU58/2 mice.

### Disturbances in folate and Hcy metabolism affect Fyn expression levels

**FIGURE 5:**
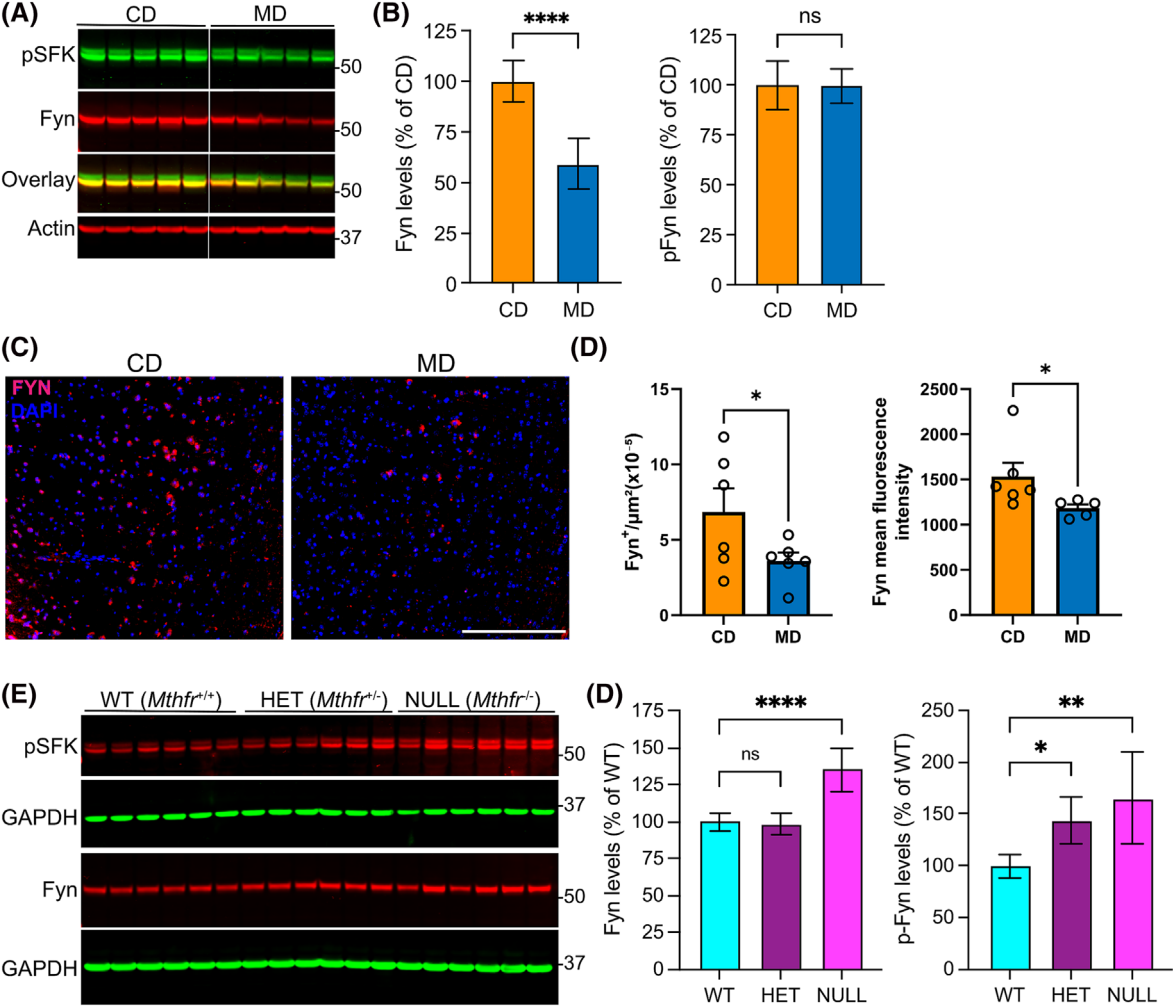
Alterations in one‐carbon metabolism modulate cortical Fyn protein expression and phosphorylation levels. (A) Cortical homogenates from control diet‐fed (CD) and methyl donor diet‐fed (MD) TAU58/2 mice were analysed for total Fyn expression levels and active Fyn using validated antibodies recognising Src family kinases phosphorylated at the regulatory Y416 site (pSFK); the lower band in the pSFK blot corresponds to phosphorylated Fyn. (B) Quantification of Fyn (left) and phosphorylated Fyn (pFyn, right). Data are mean ± *SD*, *n* = 6 mice per group; *t*‐test, ****p < 0.0001; ns, not significant. (C) Representative cortical sections from CD‐ (left) and MD‐fed (right) TAU58/2 mice, counterstained with DAPI for nuclei. Scale bars, 50 μM. (D) Quantification of total cell number normalised to the area (left) and mean fluorescence intensity (right) for Fyn in these sections. Data are mean ± SEM, *n* = 6 mice per group; *t*‐test, *p < 0.05. (E) Cortical homogenates from wild‐type *Mthfr* ^+/+^ (WT), heterozygous *Mthfr* ^+/−^ (HET) and homozygous *Mthfr* ^−/−^ (NULL) 5 week old female mice were analysed by immunoblotting for Fyn and pSFK. (F) Quantification of Fyn (left) and p‐Fyn (right) protein levels. Data (mean ± *SD*, *n* = 6 mice per group) were appraised using one‐way ANOVA with Dunnett's multiple comparison test (*F* (2, 15) = 25.73, *p* < 0.0001 for Fyn; *F* (2, 15) = 7.475, *p* = 0.0056 for pFyn); *p < 0.05, **p < 0.01, ****p < 0.0001; ns, not significant.

Aberrant tau phosphorylation has been implicated in postsynaptic recruitment of Fyn and subsequent Fyn‐mediated excitotoxicity, a key feature of tauopathies [7]. Based on the link between enhanced Fyn/p‐Tau interactions and neurodegenerative cascades in AD/FTLD‐Tau [7, 8], we next examined Fyn protein expression levels in the cortex of TAU58/2 mice. We also used antibodies recognising SFKs phosphorylated at the conserved regulatory Tyr416 (numbering depending on species) to assess potential changes in Fyn activity [46]. Western blot analyses of cortical homogenates showed that Fyn expression levels were decreased by ~40% in MD, compared with CD‐fed mice (Figure 5A,B). However, after normalising for total Fyn expression levels, no statistically significant differences in phosphorylated Fyn (p‐Fyn) levels were observed between MD and CD groups, suggesting that there was a net loss of active Fyn protein amounts in the cortex of MD, relative to CD‐fed mice. Immunostaining of cortical tissue confirmed that total Fyn protein levels were decreased by 0.4‐fold both in terms of total cell number (*p* = 0.0401) and mean fluorescence intensity (*p* = 0.0376) in MD, compared with CD‐fed mice; again, there were no changes in p‐Fyn levels among these cohorts (Figure 5C,D).

To further confirm the link between alterations in folate and Hcy metabolism and Fyn regulation, we also compared Fyn status in cortical homogenates from established mouse models of either mild (*Mthfr* ^+/−^) or severe (*Mthfr* ^−/−^) MTHFR deficiency, which recapitulate the effects of common human *Mthfr* polymorphisms [49]. Because MTHFR is a rate‐limiting enzyme in the generation of 5‐MTHF, plasma and brain levels of 5‐MTHF are expectedly reduced in MTHFR deficient mice, relative to their WT counterparts; the resulting deficit in active folate molecules leads to elevated tHcy and decreased SAM/SAH ratio [49, 50]. We have previously reported that these metabolic alterations are associated with enhanced demethylated PP2A and p‐Tau levels in brain tissue from young MTHFR‐deficient female mice [22, 45]. Additional Western blot analyses of the same cortical tissue homogenates obtained from these mice revealed that total Fyn expression levels were increased by ~35% (*p* < 0.0001) in *Mthfr* ^−/−^ relative to *Mthfr* ^+/+^ and *Mthfr* ^+/−^ mice (Figure 5E,F), indicating that Fyn proteins can become upregulated in response to severe 5‐MTHF deficiency. p‐Fyn levels were increased by ~0.4 fold (*p* = 0.04) in *Mthfr* ^+/−^ and ~0.6 fold (*p* = 0.0034) in *Mthfr* ^−/−^ mice, relative to their WT littermates (Figure 5E,F), suggesting that 5‐MTHF deficiency is associated with enhanced Fyn activity. Together, these findings provide the first in vivo evidence that Fyn expression levels are modulated by alterations in one‐carbon metabolism.

## DISCUSSION

Based on the compelling experimental, clinical and epidemiological associations between altered folate and Hcy metabolism, cognitive decline and dementia [11, 14, 51], there is growing interest in manipulating one‐carbon metabolism as a preventive or disease‐modifying approach to tauopathies. The observation that hyperhomocysteinaemia can irreversibly modify tau in an age‐dependent manner [27] further supports the rationale for an early intervention to correct one‐carbon metabolism deficiencies. In this context, previous experimental studies have introduced disturbances in one‐carbon metabolism using genetic, dietary or drug‐based means, with the goal of rescuing these disturbances with supplementation. Here, we aimed to boost cognition and reduce tau pathology in TAU58/2 mice with no other genetic alterations and no pre‐existing dietary deficits and therefore no disturbances in one‐carbon metabolism. We report that a diet enriched in L‐methylfolate, choline and betaine is effective in alleviating p‐Tau, Fyn and demethylated PP2A levels, learning and motor deficits in TAU58/2 transgenic mice that display features of both AD and FTLD‐Tau [37]. Notably, we tested a diet including L‐methylfolate rather than folic acid to overcome limitations associated with the bioconversion of folic acid to 5‐MTHF, which are common in the elderly and people with frequent genetic *Mthfr* C677T polymorphisms [52]. Those may explain why so far only high doses of folic acid have shown positive effects on cognitive decline in AD trials [51]. Moreover, our nutrient combination aimed to reduce levels of unmetabolised folic acid that can be detrimental and interfere with the transport of 5‐MTHF across the blood–brain barrier; indeed, L‐methylfolate is much more potent than folic acid in restoring CSF folate levels [53].

We chose to assess the effects of methyl donor supplements in female TAU58/2 mice due to the early onset of p‐Tau pathology and rapid development of associated symptoms, but slower disease progression than in male mice [37, 39]. Compared with their CD‐fed littermates, MD‐fed TAU58/2 mice showed faster learning in the Morris water maze and a higher level of spatial learning, as evidenced by the higher proportion of hippocampal‐dependent swim strategies used. The beneficial effects of our selected methyl donor nutrients on the learning of TAU58/2 mice are consistent with previous studies showing that supplementation with SAM can improve early cognitive deficits in AD mouse models [54]. While our custom diet improved learning, it did not affect the performance of TAU58/2 mice during testing of anxiety and disinhibition‐like behaviours. Yet, developmental dietary deficiencies in methyl donors have been associated with long‐term alterations in anxiety‐like behaviours in mice [55]. Moreover, there is a strong relationship between alterations in folate metabolism and mood disorders, and supplementation with L‐methylfolate improved depressive symptoms in clinical trials [56]. A longer dietary intervention in TAU58/2 mice may be required to detect the potential beneficial effects of our diet on anxiety‐like behaviours because those worsen with age. We speculate that longer term treatment may have a more overt effect in slowing neurodegeneration in this model given that earlier studies have shown that supplementation with folic acid for 3 years improves cognitive decline in healthy older people [57]. Additionally, long‐term (22 months) dietary supplementation with folic acid in 3 month old rats led to a marked attenuation of ageing‐induced hippocampus atrophy and cognitive decline [58]. Similarly, it would be interesting to conduct this study in older TAU58/2 mice that show more advanced neurodegeneration, based on improvements seen with folic acid supplementation in both AD patients [59] and older 3xTg AD mice [60].

Besides cognitive deficits, motor system dysfunction can be observed in FTD patients [61]. Accordingly, TAU58/2 transgenic mice display motor deficits [37]. Compared with their WT littermates, female TAU58/2 mice show progressive deficits from 2 months of age in the beam and pole tests, while changes in rotarod performance are observed only in 10 month old mice [37], hence not used here. We found that MD‐fed mice performed much better in both the beam and pole tests. These significant improvements could not be attributed to changes in mouse body weights, which were similar in both CD and MD groups over the experimental period.

The pathological accumulation of p‐Tau, the defining feature of tauopathies [3], is recapitulated in TAU58/2 mice [37]. Our findings show that supplementation with methyl donors is effective in reducing p‐Tau at the pathological CP13 and PHF‐1 phosphoepitopes in the cortex of TAU58/2 mice. Based on the prevalent role of methylated PP2A holoenzymes in dephosphorylating p‐Tau [5], decreased levels of p‐Tau at the PHF‐1 and CP13 epitopes could directly result from enhanced PP2A methylation in MD‐ vs CD‐fed mice. In line with these findings, folic acid decreases pS396‐Tau levels by enhancing PP2A methylation in diabetic mice [62].

We provide the first in vivo evidence that supplementation with methyl donors can reduce Fyn protein expression levels. This is highly significant because several studies point to the critical role of Fyn in the neurodegenerative cascades underlying tauopathies [7]. Deletion of Fyn in tauopathy mouse models decreases cortical levels of pS199/S202‐Tau [63] and pS202/T205‐Tau [64]. Likewise, Fyn inhibition reduces p‐Tau at the pS202/T205 and PHF‐1 epitopes and rescues cognitive deficits in a P301S‐Tau model [65]. Thus, the reduction in cortical Fyn protein levels could mediate the decrease in p‐Tau at the S202 and PHF‐1 epitopes and amelioration of cognitive deficits observed in our MD‐fed TAU58/2 mice. In support of this hypothesis, severe MTHFR deficiency, which is associated with enhanced tau phosphorylation at the PHF‐1 epitope [22], promoted an increase in Fyn activity and protein levels in *Mthfr* ^−/−^ mice.

We have reported that altered PP2A methylation, AD‐like tau phosphorylation and FTLD‐Tau mutations (including the P301S mutation) all decrease the binding of PP2A to tau. This interferes with the ability of PP2A to dephosphorylate tau while enhancing the p‐Tau‐Fyn interactions that mediate excitotoxicity in tauopathies [34]. Alterations in one‐carbon metabolism and PP2A methylation also affect Fyn subcellular targeting and function in N2a cells [46]. Altogether, these findings support the hypothesis that disturbances in one‐carbon metabolism can promote the accumulation of pathological tau by deregulating normal PP2A/tau/Fyn interactions. Besides affecting methylation‐sensitive targets, our diet likely exerts additional beneficial effects in TAU58/2 mice. It decreases tHcy levels, which can promote excitotoxicity and oxidative stress [11], and enhances antioxidant capacity, via increasing GSH and 5‐MTHF levels. Based on the known link between neuroinflammation and neurodegeneration and previous clinical trials showing a reduction in peripheral cytokines in response to folic acid in mild cognitive impairment (MCI) [66] and early AD patients [59], it would be interesting in future studies to comprehensively assess changes in neuroinflammatory markers in our mice. The choline‐mediated increase in Ach levels could also improve learning processes in MD‐fed mice. However, cholinergic deficits that are prevalent in AD patients and mouse models are not a major feature of FTLD‐Tau patients and transgenic mouse models of FTD [67].

## CONCLUSION

While AD and FTLD‐Tau are prevalent forms of dementia, there are currently no preventive or disease‐modifying treatments for these disorders. In the present study, we show that food supplementation with L‐methylfolate/choline/betaine can alleviate p‐Tau pathology and associated learning and motor deficits in a tauopathy mouse model. Here, we provide the first in vivo evidence for a link between folate metabolism and the regulation of Fyn, a tyrosine kinase that plays a central role in normal homeostasis and tau‐mediated neurodegenerative processes [68]. We propose that methyl donor supplements can decrease cortical pathological p‐Tau levels, at least in part by decreasing Fyn levels and enhancing the methylation of PP2A, the major brain tau phosphatase.

Because tau pathology precedes symptomatic cognitive deficits by many years [69] and is linked to mild behavioural impairment in preclinical AD [70], an early intervention targeting nutrient‐dependent risk factors such as deficiencies in one‐carbon metabolism could provide a safe and plausible option to decrease the onset and progression of p‐Tau pathology. Our experimental findings provide support for conducting novel trials with L‐methylfolate/choline/betaine—rather than folic acid—to prevent or mitigate AD/FTD.

## AUTHOR CONTRIBUTIONS

ES and JS conceptualised the study and designed the diet. ES, JS, LI, YK, AvH, and GT contributed to the design of animal experiments and data interpretation. AvH performed all animal experiments, tissue collection and behavioural testing and analyses. AF performed all histology experiments and related analyses. GT and JS performed all Western blot and related analyses. AvH and ES wrote the manuscript with input from all authors. All authors read and approved the final manuscript.

## CONFLICT OF INTEREST STATEMENT

None.

## ETHICS STATEMENT

All experiments were approved by the Macquarie University Animal Care and Ethics Committee.

## Supporting information


**Figure S1.** Dietary supplementation with methyl donors does not affect the performance of TAU58/2 mice in the elevated plus maze and open field tests or swim speed in the Morris water maze. A. There are no statistically significant differences in time in open or closed arms in the elevated plus maze (EPM) in TAU58/2 mice fed either the control (CD) or methyl donor (MD) diets. B. There are no statistically significant differences in time spent in inner or outer zones of the open field (OF) arena between MD and CD fed TAU58/2 mice. C. There are no statistically significant differences in distance travelled over a 10‐minute period in the OF arena between MD and CD fed TAU58/2 mice. D. There was no difference in mean swim speed in the Morris water maze (MWM). All graphs in A‐D show mean ± SEM; *n* = 12 mice/diet group.
**Figure S2.** Dietary supplementation with methyl donors does not affect cortical tau phosphorylation at pS214 and pS422 in TAU58/2 mice. A. Representative images of immunofluorescent staining (left panel) of pS422‐Tau in cortical regions of TAU58/2 mice fed either a control diet (CD) or methyl donor diet (MD); slides were counterstained with DAPI to label nuclei. There are no statistically significant differences after quantification of total cell number normalised to area (middle panel) and mean fluorescence intensity (right panel) for pS422. B. Representative images of immunofluorescent staining (left panel) of pS214 in cortical regions of CD and MD fed TAU58/2 mice, counterstained with DAPI for nuclei. There are no statistically significant differences after quantification of total cell number normalised to area (middle panel) and mean fluorescence intensity (right panel) for pS214‐Tau. All graphs in A‐B show mean ± SEM, *n* = 6 mice/diet group. Scale bar, 50 μM.

## Data Availability

The data that support the findings of this study are available from the corresponding author upon reasonable request.
