# Supplementary material for: Methyl donor supplementation reduces phospho‐Tau, Fyn and demethylated protein phosphatase 2A levels and mitigates learning and motor deficits in a mouse model of tauopathy
Source: Neuropathol Appl Neurobiol. 2023 Aug 28;49(4):e12931. doi: 10.1111/nan.12931 (PMC10947299; doi:10.1111/nan.12931)
Supplement: Supplementary file 1 — Figure S1. Dietary supplementation with methyl donors does not affect the performance of TAU58/2 mice in the elevated plus maze and open field tests or swim speed in the Morris water maze. A. There are no statistically significant differences in time in open or closed arms in the elevated plus maze (EPM) in TAU58/2 mice fed either the control (CD) or methyl donor (MD) diets. B. There are no statistically significant differences in time spent in inner or outer zones of the open field (OF) arena between MD and CD fed TAU58/2 mice. C. There are no statistically significant differences in distance travelled over a 10‐minute period in the OF arena between MD and CD fed TAU58/2 mice. D. There was no difference in mean swim speed in the Morris water maze (MWM). All graphs in A‐D show mean ± SEM; n = 12 mice/diet group. Figure S2. Dietary supplementation with methyl donors does not affect cortical tau phosphorylation at pS214 and pS422 in TAU58/2 mice. A. Representative images of immunofluorescent staining (left panel) of pS422‐Tau in cortical regions of TAU58/2 mice fed either a control diet (CD) or methyl donor diet (MD); slides were counterstained with DAPI to label nuclei. There are no statistically significant differences after quantification of total cell number normalised to area (middle panel) and mean fluorescence intensity (right panel) for pS422. B. Representative images of immunofluorescent staining (left panel) of pS214 in cortical regions of CD and MD fed TAU58/2 mice, counterstained with DAPI for nuclei. There are no statistically significant differences after quantification of total cell number normalised to area (middle panel) and mean fluorescence intensity (right panel) for pS214‐Tau. All graphs in A‐B show mean ± SEM, n = 6 mice/diet group. Scale bar, 50 μM. [file NAN-49-0-s001.docx]

Methyl donor supplementation reduces phospho-Tau, Fyn and demethylated PP2A levels, and mitigates learning and motor deficits in a mouse model of tauopathy

**SUPPLEMENTARY MATERIAL**

Supplementary Fig. S1

Supplementary Fig. S2

Supplementary Methods: Detailed protocol for plasma metabolite analyses

**
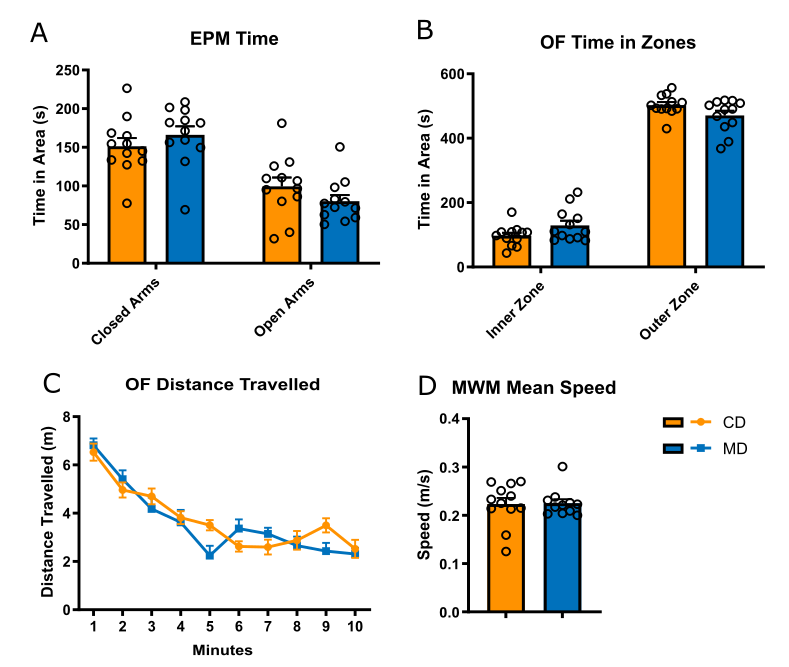
**

**Fig. S1. Dietary supplementation with methyl donors does not affect the performance of TAU58/2 mice in the elevated plus maze and open field tests or swim speed in the Morris water maze. A.** There are no statistically significant differences in time in open or closed arms in the elevated plus maze (EPM) in TAU58/2 mice fed either the control (CD) or methyl donor (MD) diets. **B.** There are no statistically significant differences in time spent in inner or outer zones of the open field (OF) arena between MD and CD fed TAU58/2 mice. **C.** There are no statistically significant differences in distance travelled over a 10-minute period in the OF arena between MD and CD fed TAU58/2 mice. **D.** There was no difference in mean swim speed in the Morris water maze (MWM). All graphs in **A-D** show mean ± SEM; *n* =12 mice/diet group.

**Fig. S2. Dietary supplementation with methyl donors does not affect cortical tau phosphorylation at pS214 and pS422 in TAU58/2 mice. A.** Representative images of immunofluorescent staining (left panel) of pS422-Tau in cortical regions of TAU58/2 mice fed either a control diet (CD) or methyl donor diet (MD); slides were counterstained with DAPI to label nuclei. There are no statistically significant differences after quantification of total cell number normalised to area (middle panel) and mean fluorescence intensity (right panel) for pS422. **B.** Representative images of immunofluorescent staining (left panel) of pS214 in cortical regions of CD and MD fed TAU58/2 mice, counterstained with DAPI for nuclei. There are no statistically significant differences after quantification of total cell number normalised to area (middle panel) and mean fluorescence intensity (right panel) for pS214-Tau. All graphs in **A-B** show mean ± SEM, *n* = 6 mice/diet group. Scale bar, 50 µM.

**Detailed protocol for plasma metabolite analyses**

100µl plasma was pre-treated with 50µl of 1mM ascorbic acid and 350µl acetonitrile, vortexed, sonicated and centrifuged for 15 min at 15,000 rpm, and the supernatant used for analysis. To quantify amino compounds, 50µl of supernatants or serially diluted mixed standard solutions of amino acids including taurine was mixed with 50µl of their 13C- or D-labelled internal standards, 100µl of a dansyl chloride solution and 50µl of borate buffer and incubated for 30 min at 40^o^C. 10µl was injected to run UPLC-MRM/MS for quantitation. Standard substance calibration curves were constructed with internal calibration. Concentrations of the metabolites were calculated with the measured analyte to internal standard peak area ratios by interpolating the linear regression calibration curves. To quantify folate metabolites, samples were diluted 10 times in water and loaded onto polymeric reversed-phase solid-phase extraction (SPE) cartridges (60mg/1mL), which were activated with methanol and conditioned with water before use, on a 48-positive SPE manifold. Under a 2-psi positive-pressure pressure, the resin retained metabolites were eluted with methanol and dried under gas flow in a nitrogen evaporator. The residues were reconstituted in 50µl of 20% methanol. 20µl was analysed by MRM/MS using a C18 UPLC column (2.1 × 150mm, 1.8µm) for gradient elution, with water-acetonitrile containing 0.1% formic acid as the mobile phase. Calibration curves were constructed from serially diluted standard solutions of the targeted, and concentrations of the metabolites were calculated with the measured peak areas by interpolating the linear-regression calibration curves. For quantification of other metabolites, 200µl sample were mixed with 50µl SAH-D4 (internal standard), 100µl 1mM ascorbic acid and 300µl chloroform, vortexed and centrifuged for 5 min at 15,000rpm at 10^o^C. The aqueous phase was dried under gas flow in a nitrogen evaporator. Residues were reconstituted in 50µl aqueous acetonitrile and 10µl was injected onto a HILIC UPLC column (2.1 x 100mm, 1.7µm) to run LC-MRM/MS. Calibration curves were constructed using serially diluted standard solutions and concentrations of the metabolites were calculated with the measured analyte to internal standard (SAH-D4) peak area ratios by interpolating the linear regression calibration curves.
